# Menstrual psychosis diagnosis: Does it still hold?

**DOI:** 10.1192/j.eurpsy.2022.512

**Published:** 2022-09-01

**Authors:** C. Adão, I. Donas-Boto, A. Velosa, P. Trindade, R. Caetano

**Affiliations:** 1 Centro Hospitalar de Lisboa Ocidental, Psychiatry Department, Lisboa, Portugal; 2 NOVA Medical School, Psychiatry And Mental Health, Lisboa, Portugal

**Keywords:** brief psychotic disorder, Psychosis, menstrual, catamenial

## Abstract

**Introduction:**

Menstrual psychosis was first described in the 18th century. Brockington defined its characteristics: acute onset; brief duration with full recovery; confusion, stupor and mutism, delusions, hallucinations, or a manic syndrome and periodicity in temporal association with the menstrual cycle.

**Objectives:**

Description of a clinical case of menstrual psychosis and review of the literature.

**Methods:**

Description of a clinical case. Non systematic review of the literature, searching the terms “psychosis”; “menstrual”; “catamenial” in the databases Pubmed, Medline and Cochrane.

**Results:**

Female, 39-year-old patient. No psychiatric history until the postpartum period of a traumatic vaginal birth, when she developed stupor and mutism which lasted for two days. During the following 2 years, she progressively presented with sadness, asthenia, anhedonia, insomnia and incapacity for self-care. She was prescribed paroxetine and olanzapine, with partial recovery. Subsequently, she had at least 6 episodes with about 3-day duration of asthenia, food refusal, insomnia, incapacity for self-care, disorganization of thought and behavior and mystical and persecutory delusions, coincident with the beginning of menstruation. She was hospitalized in two of them and received treatment with venlafaxine 75mg and paliperidone 6mg, with psychotic symptoms remission after a week.

**Conclusions:**

This case presents the characteristics of menstrual psychosis. This is a rare condition, with only 30 reported cases worldwide. According to current classification systems, this condition fulfills diagnostic criteria for brief psychotic disorder. Nonetheless, studying in more detail this disorder could be interesting, with the goal of deepening the knowledge of the neurobiology of psychosis, particularly the effects of estrogen on this disorder.

**Disclosure:**

No significant relationships.

